# Supplementary material for: Transcriptomic Insights into Mechanisms of Early Seed Maturation in the Garden Pea (Pisum sativum L.)
Source: Cells. 2020 Mar 23;9(3):779. doi: 10.3390/cells9030779 (PMC7140803; doi:10.3390/cells9030779)
Supplement: Supplementary file 1 [file cells-09-00779-s001.zip › Supplementary materials/FileS1.tar.gz!/FileS1/short_report.pdf]

Short report

	contigs_filtered_cdhit
Genes	44756
Avg. number of exons per isoform	4.9
Transcripts	25756
Transcripts > 500 bp	23199
Transcripts > 1000 bp	17693
Aligned	25702
Uniquely aligned	24336
Multiply aligned	172
Unaligned	54
Avg. aligned fraction	0.983
Avg. alignment length	1576.27
Avg. mismatches per transcript	8.804
Misassemblies	608
Database coverage	0.283
Duplication ratio	1.056
50%-assembled genes	13502
95%-assembled genes	4313
50%-covered genes	13864
95%-covered genes	4493
50%-assembled isoforms	13755
95%-assembled isoforms	4338
50%-covered isoforms	14120
95%-covered isoforms	4520
Mean isoform coverage	0.794
Mean isoform assembly	0.779
Predicted genes	17015
50%-matched	10839
95%-matched	5965
Unannotated	0.645

generated by rnaQUAST

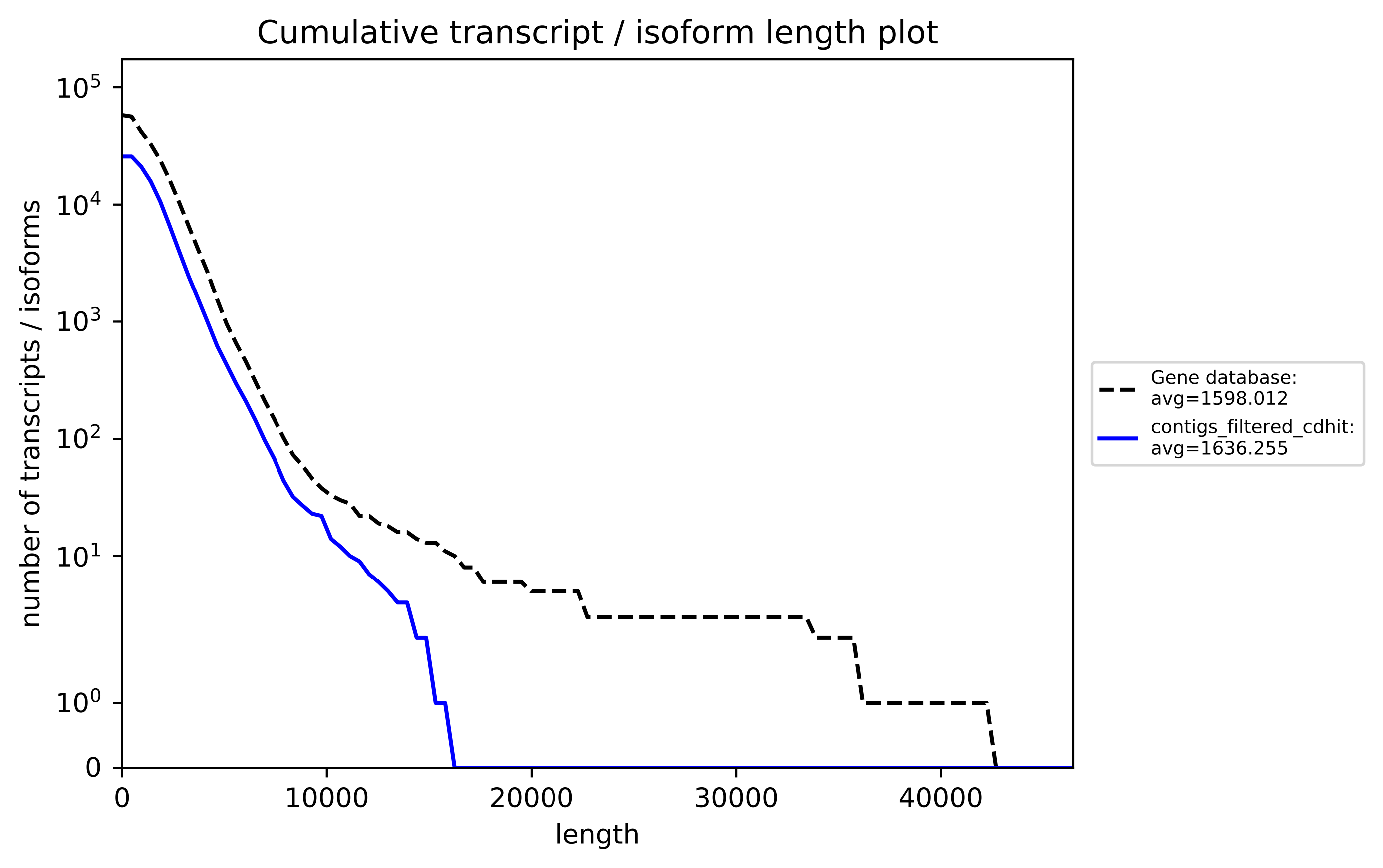
Cumulative transcript / isoform length plot
number of transcripts / isoforms
length
10^5
10^4
10^3
10^2
10^1
10^0
0
0
10000
20000
30000
40000
Gene database:
avg=1598.012
contigs_filtered_cdhit:
avg=1636.255

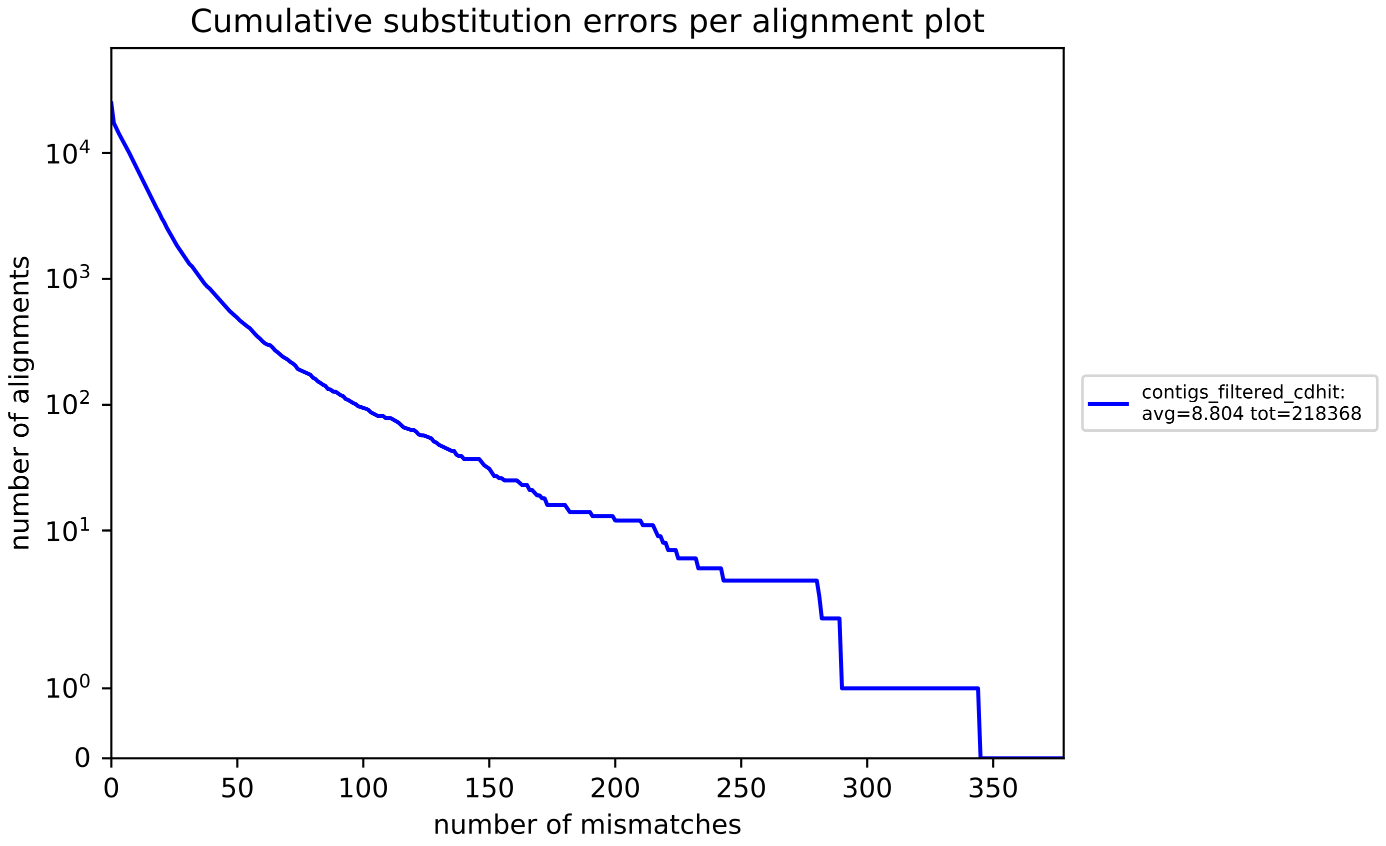

Cumulative substitution errors per alignment plot
number of alignments
number of mismatches
10^4
10^3
10^2
10^1
10^0
0
0
50
100
150
200
250
300
350
contigs_filtered_cdhit:
avg=8.804 tot=218368

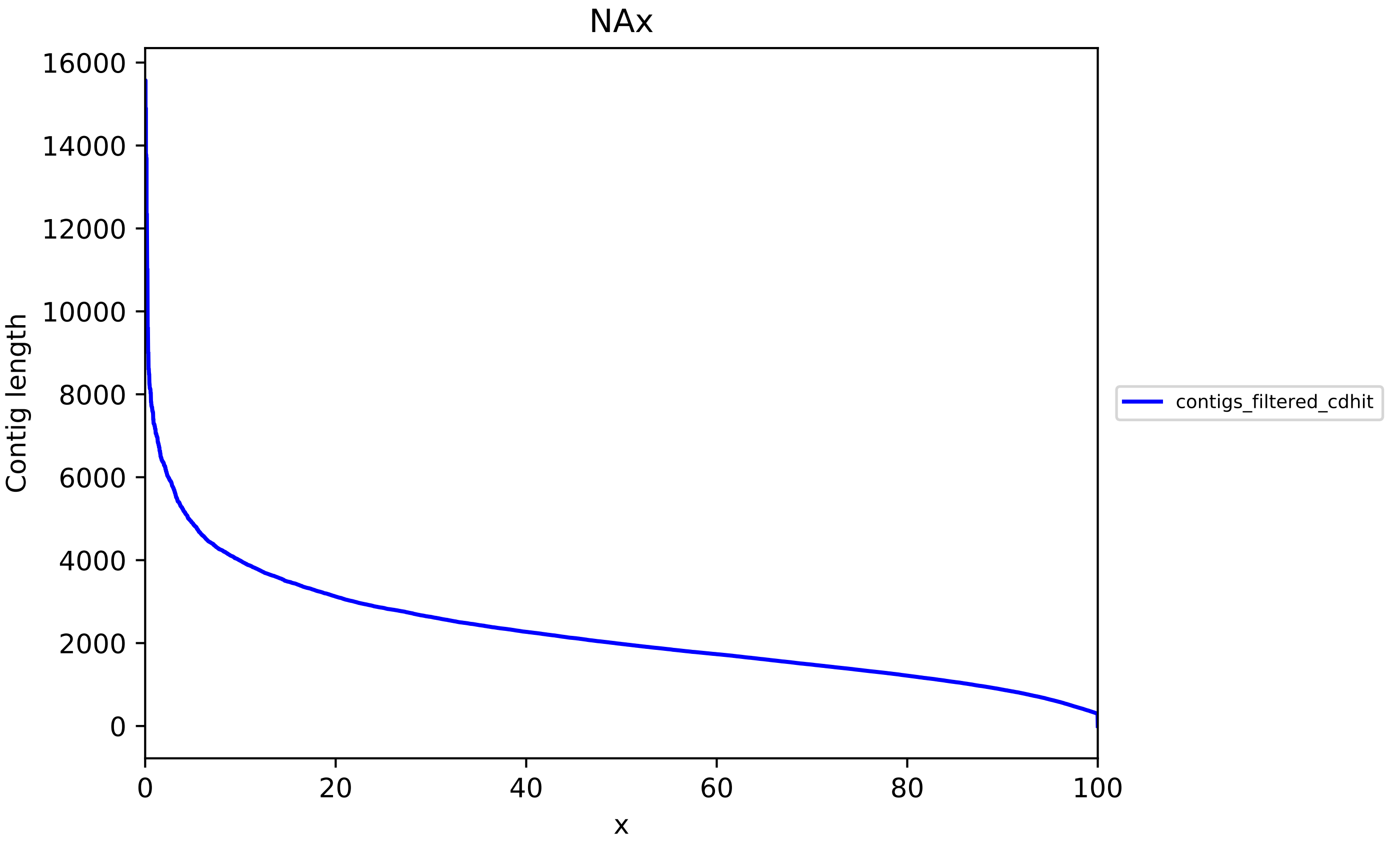
NAx
16000
14000
12000
10000
8000
6000
4000
2000
0
Contig length
0
20
40
60
80
100
x
contigs_filtered_cdhit

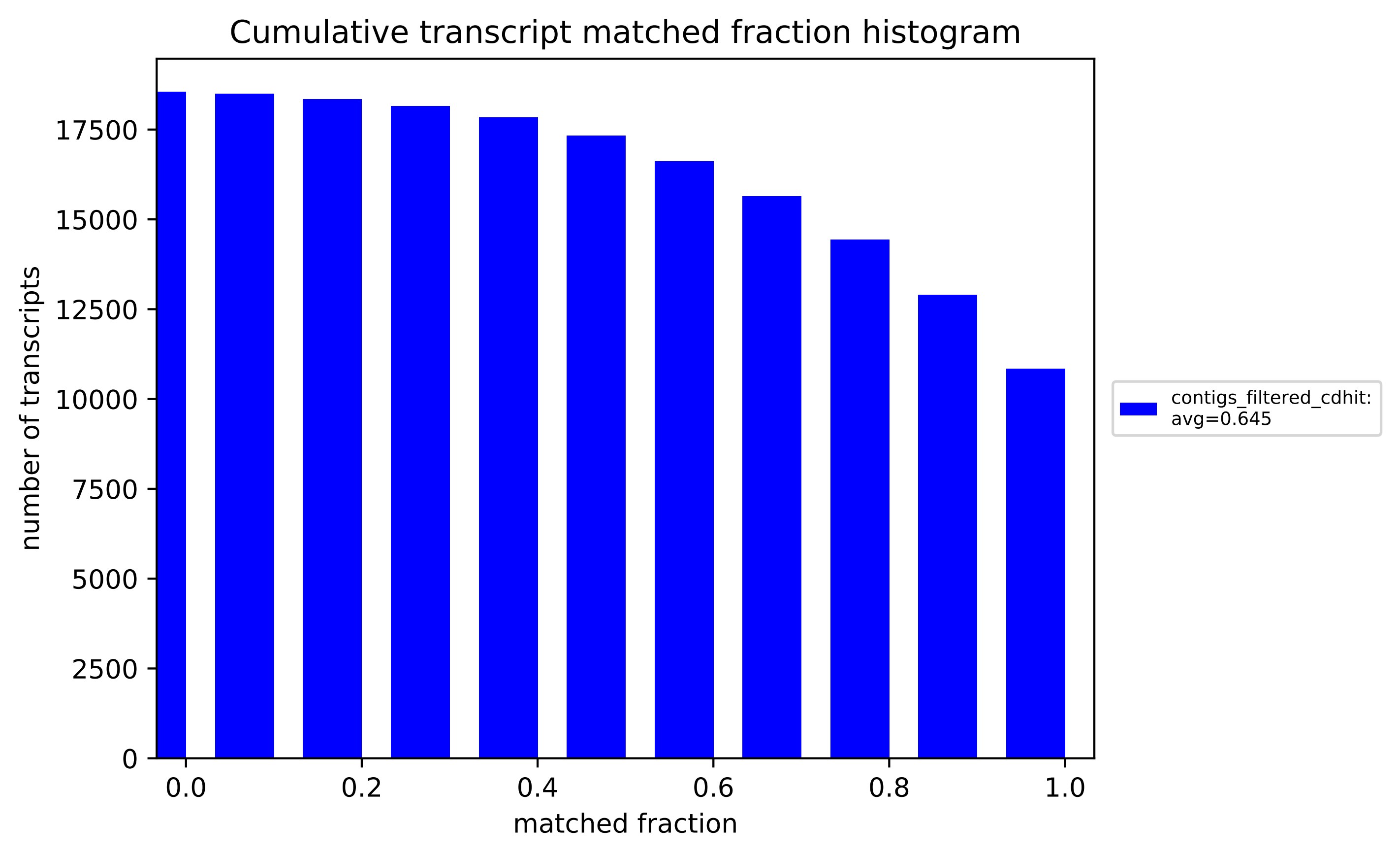

Cumulative transcript matched fraction histogram
number of transcripts
0
2500
5000
7500
10000
12500
15000
17500
0.0
0.2
0.4
0.6
0.8
1.0
matched fraction
contigs_filtered_cdhit:
avg=0.645

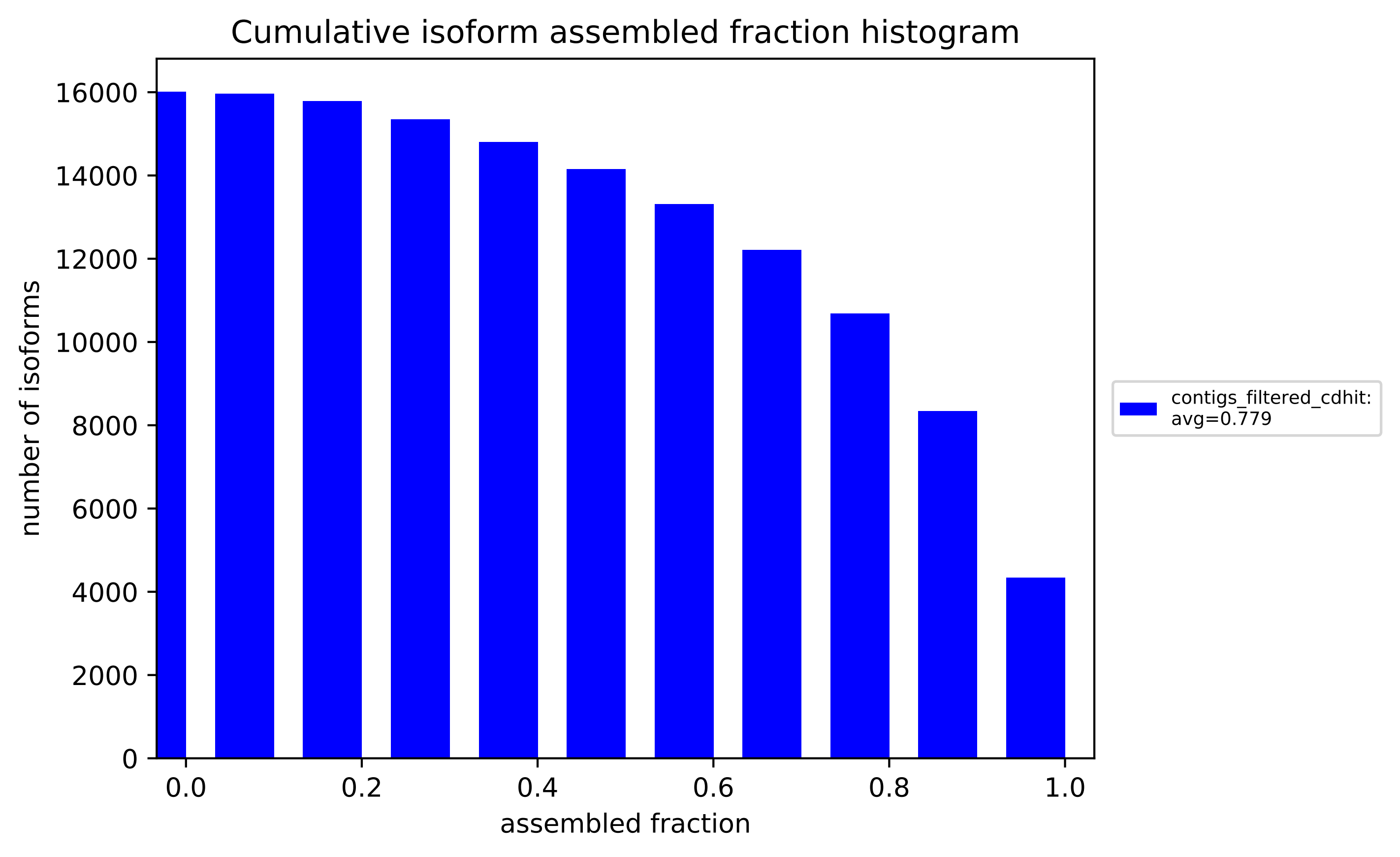
Cumulative isoform assembled fraction histogram
number of isoforms
0
2000
4000
6000
8000
10000
12000
14000
16000
0.0
0.2
0.4
0.6
0.8
1.0
assembled fraction
contigs_filtered_cdhit:
avg=0.779

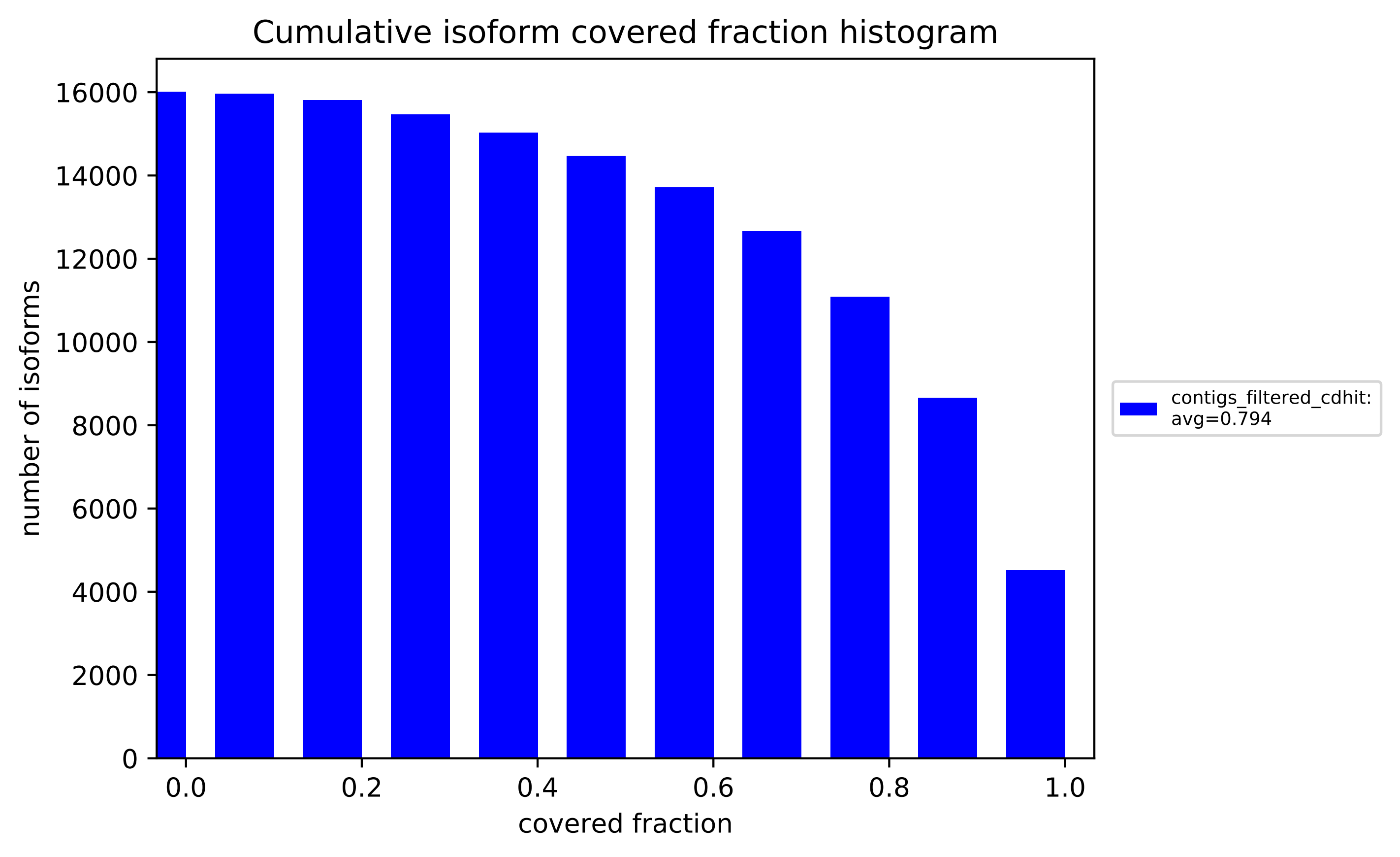

Cumulative isoform covered fraction histogram
number of isoforms
covered fraction
0
2000
4000
6000
8000
10000
12000
14000
16000
0.0
0.2
0.4
0.6
0.8
1.0
contigs_filtered_cdhit:
avg=0.794
